# Neuropathology of Parkinson’s disease after focused ultrasound thalamotomy

**DOI:** 10.1038/s41531-022-00319-6

**Published:** 2022-05-12

**Authors:** Shunsuke Koga, Mariam Ishaque, W. Jeffrey Elias, Binit B. Shah, Aya Murakami, Dennis W. Dickson

**Affiliations:** 1 Department of Neuroscience, Mayo Clinic, Jacksonville, FI USA; 2 Department of Neurosurgery, University of Virginia Health Science Center, Charlottesville, VI USA

**Keywords:** Parkinson's disease, Neuroscience

## Abstract

Focused ultrasound (FUS) thalamotomy is an emerging treatment for tremor-dominant Parkinson’s disease (PD). We report the first postmortem neuropathologic study of FUS thalamotomy in a 68-year-old man with tremor-dominant PD, which was performed seven months before he died. Although the peak voxel temperature at the target was <54 °C, his tremor improved on intraoperative and postoperative assessments. Additionally, postoperative MRI demonstrated a thalamic lesion. Lewy body-related pathology consistent with PD was detected. There was also a 5-mm lesion in the ventral lateral thalamus characterized by demyelination and neuropil loss, with many lipid-laden macrophages, but no lymphocytic infiltrates and relatively preserved neurons and axons. Additional pathological assessments after FUS thalamotomy are needed to determine if the observed brain changes are typical of this procedure.

## Introduction

Parkinson’s disease (PD) is the most common neurodegenerative movement disorder characterized by bradykinesia, rigidity, postural instability, and tremor at rest^1^. Dopamine replacement therapy is the gold standard treatment for PD, but there is a subset of tremor-dominant PD patients for whom medical therapy does not achieve successful tremor control. Deep brain stimulation (DBS) targeting the subthalamic nucleus, globus pallidus interna and ventral intermediate nucleus of the thalamus has become widely used^2^. DBS requires an open cranial procedure for device implantation, which is associated with the risk of hemorrhagic and infectious complications^3,4^.

Magnetic resonance (MR)-guided focused ultrasound (FUS) is an incision-free procedure for precise thermal ablation of deep structures in the brain^5^. Clinical trials of FUS thalamotomy targeting the ventral intermediate nucleus of the thalamus in patients with refractory essential tremor have shown safety and efficacy. This led to approval of this treatment by the United States Food and Drug Administration (FDA) in 2016^6–8^. Subsequently, FUS thalamotomy was approved in 2018 by the FDA for tremor-dominant PD intolerant or refractory to dopamine replacement therapy^9^. Very recently, FUS pallidotomy was also approved for treatment of dyskinesia in PD^10^. Longitudinal changes in lesions after FUS treatment have been investigated with MRI, which show changes consistent with necrosis in the center of the lesion and cytotoxic and vasogenic edema in the periphery^11,12^. Histopathological correlates of these lesions have not been reported in humans, only in experimental animals^13–15^. We report the first postmortem neuropathological findings of a PD patient who underwent FUS thalamotomy.

## Results

### Case report

The patient was a 68-year-old Caucasian right-handed man with a 12-year history of tremor-dominant PD. His symptoms started with a left foot tremor at 56 years of age. By age 67, the tremor was bilateral, and it impaired activities of daily living. Carbidopa/levodopa (400–700 mg/day) suppressed his tremors but produced severe dyskinesias. On neurological examination, he had bradykinesia and bilateral tremor, most severe in the left lower and right upper extremities. Additional symptoms included falls and daytime visual hallucinations. Neurocognitive testing showed no cognitive impairment.

His right hand tremor was the most bothersome symptom and was not optimally controlled with medications. He was a candidate for either DBS implantation or a clinical trial of FUS subthalamotomy^16^. After the discussion of the merits and limitations of each procedure, he opted for FUS subthalamotomy as he perceived it to be less invasive. He underwent a screening head CT scan, which showed a favorable skull density ratio (SDR; a ratio of cortical to cancellous bone) of 0.55, but also revealed a right intraocular mass. Thus, he was excluded from the FUS subthalamotomy clinical trial^16^. Further workup indicated malignant choroidal melanoma with metastases to the liver (stage IV). He underwent right eye enucleation and adjuvant chemotherapy. After the treatment for his melanoma, he underwent a left FUS thalamotomy procedure to control disabling right upper extremity tremor.

**Fig. 1:**
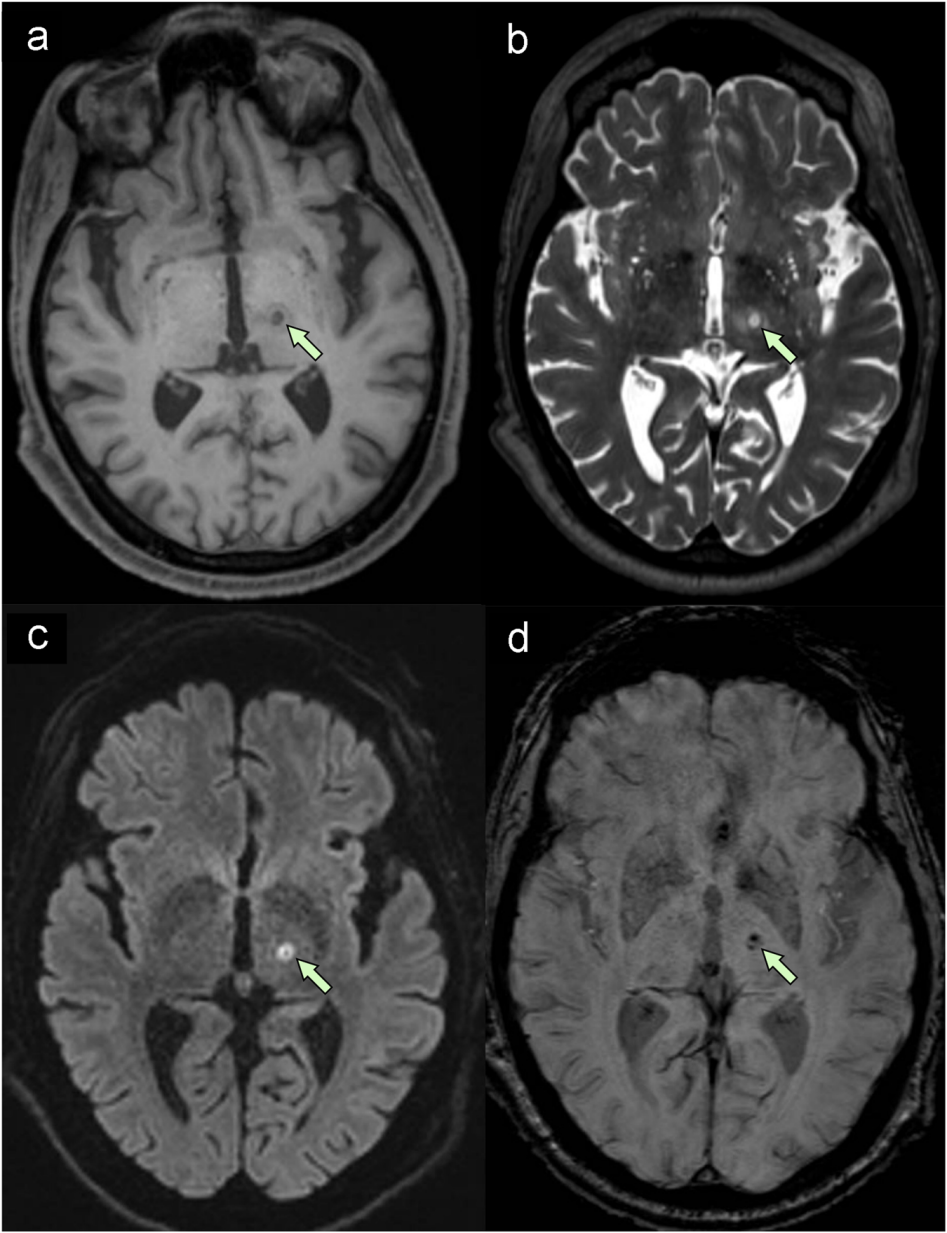
Representative MRI on postoperative day 1. **a** T1-weighted image shows hypointense lesion in the thalamus. **b** T2-weighted image shows hyperintense lesion with a small hypointense core. **c** Diffusion-weighted image demonstrates diffusion restriction within the lesion, suggesting tissue infarction. **d** Susceptibility-weighted image demonstrates hypointense blood products within the lesion.

Intraoperative SDR was 0.51. He demonstrated improved intra- and postoperative tremor control without any adverse events. An MRI of the brain on postoperative day 1 demonstrated an expected thalamic lesion with surrounding edema, restricted diffusion and intralesional blood products (Fig. 1). Two months after the thalamotomy, he was hospitalized with progressive tetraparesis and paresthesias while undergoing immunotherapy treatment for his melanoma. He died at home on hospice care 7 months after FUS thalamotomy.

### MR-guided FUS treatment characteristics

The patient received eleven transcranial sonications. The first five sonications were significantly modulated to avoid acoustic cavitation (i.e., the collapse of bubbles); however, cavitation was observed during the alignment phase. To mitigate this, the multi-echo imaging was turned off, and additional no-pass regions were designated to the membrane folds. The water bath was drained and refilled, and the tissue type was changed for the sensitivity of the cavitation detectors. Three alignment sonications were then performed, requiring 6700 J of energy for a mild temperature rise at the focus. This ensured that the natural focus of the transducer matched the two-dimensional plane of thermal imaging. Finally, three therapeutic sonications delivered acoustic energies of 16,000, 24,000, and 36,000 J. The peak voxel temperatures at the target only reached 51–54 °C, but the sonication durations were prolonged (20–35 s), and the tremor was gone in both resting and postural phases.

### Pathological findings

**Fig. 2:**
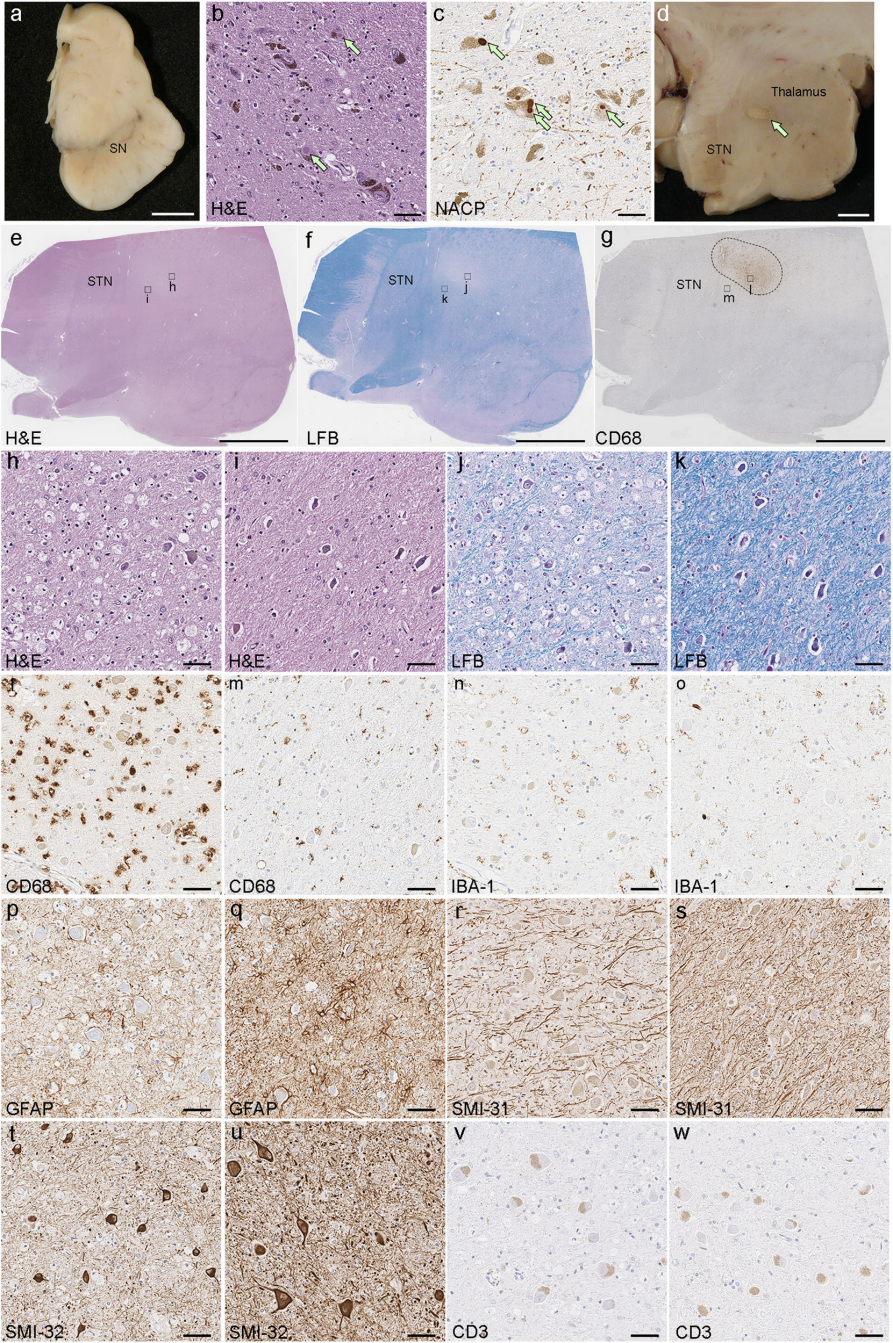
Macroscopic and histopathologic findings of the patient. **a** Decreased neuromelanin pigment in the substantia nigra. **b** The substantia nigra shows severe neuronal loss with gliosis and extracellular neuromelanin. The remaining neurons contain Lewy bodies (arrows). **c** Immunohistochemistry for α-synuclein (NACP antibody) reveals abundant Lewy bodies (arrows) and Lewy neurites in the substantia nigra. **d** A lesion related to FUS thalamotomy is visible in the ventral lateral thalamus at the level of the mammillothalamic tract. **e**–**m** Lower magnification of the thalamus on hematoxylin and eosin (H&E) stains (**e**), Luxol fast blue-periodate-Schiff stain (LFB-PAS) stains (**f**), and immunohistochemistry for CD68 (**g**) show a 5-mm × 3-mm lesion in the ventral lateral thalamus (dotted ellipse in **g**). Boxes in **e**, **f**, and **g** indicate locations of high magnification images in the lesion (**h**, **j**, and **l**) and the adjacent area (**i**, **k**, and **m**). H&E stains show abundant foamy macrophages in the lesion (**h**), but the neuronal population is comparable to the adjacent area (**i**). LFB-PAS stains show demyelination in the lesion (**j**), while the adjacent area has preserved myelination (**k**). The lesion has abundant CD68-positive foamy macrophages (**l**) with a paucity of IBA-1-positive macrophages (**n**), while the adjacent area has sparse macrophages (**m**, **o**). Immunohistochemistry for GFAP shows reduced immunoreactivity in the macrophage-rich region (**p**) compared with the adjacent area (**q**). Immunohistochemistry for SMI-31 shows relatively intact axons in the lesion (**m**) compared to the adjacent area (**s**). Immunohistochemistry for SMI-32 shows preserved neuronal populations in both the lesion (**t**) and the adjacent area (**u**). There is no infiltration of T cells on CD3 staining in either region (**v**, **w**). Scale bars: 5 mm in **a** and **d**–g 50 μm in **b**, **c** and **h**–**w**. SN substantia nigra, STN subthalamic nucleus.

The fixed left hemibrain weighed 600 g. Macroscopic findings revealed no significant cortical atrophy and no enlargement of frontal or temporal horns of the lateral ventricle. The hippocampus, amygdala, basal ganglia, and subthalamic nucleus were unremarkable. The substantia nigra (Fig. 2a) and locus coeruleus had decreased neuromelanin pigment. Haematoxylin and eosin (H&E) stains showed severe neuronal loss with gliosis and extracellular pigment, as well as Lewy bodies (arrows) in the remaining neurons in the substantia nigra (Fig. 2b). Immunohistochemistry for α-synuclein revealed abundant Lewy bodies and Lewy neurites (Fig. 2c), consistent with the neuropathological diagnosis of PD^17^.

A 5-mm × 3-mm lesion was observed in the ventral lateral thalamus at the level of the mammillothalamic tract (arrow in Fig. 2d). The lesion was characterized by many foamy macrophages, immunoreactive for CD68 and less for IBA-1, but relatively preserved neurons compared to the adjacent thalamus (Fig. 2e–o). There was myelin loss and a few myelin figures in the macrophages with Luxol fast blue-periodate-Schiff (LFB-PAS) stain (Fig. 2i). There were reactive astrocytes throughout the thalamus, but GFAP immunoreactivity was decreased in the macrophage-rich region (Fig. 2p, q). Immunohistochemistry for phosphorylated neurofilament (SMI-31) showed relatively preserved axons, and non-phosphorylated neurofilament (SMI-32) showed relatively preserved neuronal populations in this lesion (Fig. 2r–u). There were no axonal swellings on amyloid precursor protein (APP) immunohistochemistry. Immunohistochemistry for CD3 (Fig. 2v, w), CD45RO and CD20 revealed no significant infiltration of T cells or B cells in the lesion or adjacent tissue. No infarction or hemorrhage was observed. Additional neuropathologic findings included sparse neurofibrillary tangles in the entorhinal cortex, but no senile plaques consistent with primary age-related tauopathy (Braak neurofibrillary tangle stage II; Thal amyloid phase 0).

## Discussion

We describe the first neuropathologic findings of a patient with PD who underwent MR-guided FUS thalamotomy. The clinical endpoint of tremor suppression was achieved, and postoperative MRI indicated an expected ablation lesion in the thalamus. The postmortem neuropathological assessment showed brainstem-predominant Lewy body disease and moderate to marked neuronal loss in the ventral lateral substantia nigra consistent with PD^17^. A 5-mm lesion in the ventral lateral thalamus consistent with a FUS-induced ablation was observed. The lesion was characterized by demyelination and neuropil loss, with many lipid-laden macrophages and relatively preserved neurons and axons.

The desired effect of high-intensity FUS is considered focal tissue necrosis due to the thermal effect^5^. Experimental animal models demonstrate necrosis within 5–10 s with temperatures above 54 °C or 10–100 s at temperatures between 50 and 54 °C^18,19^. SDR is the most critical determinant for peak temperature in lesions^19,20^, and it positively correlates with maximal temperature^21^. In a recent study by Yang and colleagues, all subjects with an SDR ≥ 0.45 reached peak temperatures of ≥54 °C, while 28.6% of patients with an SDR < 0.45 did not reach this target temperature^19^. Our patient had an SDR of 0.55 predicted by preoperative CT scan, but the temperature at the ablation focus had a peak of only 54 °C with higher than expected acoustic energies and prolonged sonication durations. It remains unknown why the observed peak temperature was below 55 °C despite the favorable SDR, but other factors, such as the number of cavitation halts^22^, may have hindered the temperature rise. Importantly, the clinical endpoint of tremor suppression was achieved; therefore, further attempts to increase peak temperature at the target with increasing energies and longer sonication durations were not made, and an adequate thalamic lesion was expected.

Consistent with previous studies, postoperative MRI demonstrated a T2 hypointense thalamic lesion with restricted diffusion, suggestive of necrosis^11^. The lesion observed at autopsy lacked significant neuronal loss, and no findings suggestive of infarction or necrosis. Several studies have reported variability in lesion appearance on MRI after FUS thalamotomy, which may associate with clinical efficacy^11,12^. None have had neuropathologic assessments. In a prospective study on seven patients with essential tremor, FUS lesions were visible as T2 hypointense or T2 hyperintense lesions one year after the procedure in six patients. In contrast, the lesion completely disappeared after 1 year in one patient with the lowest clinical improvement in the Clinical Rating Scale for Tremor^12^. It remains to be determined whether FUS thalamotomy can induce necrosis with peak temperatures of 54 °C or greater, and how this correlates with short- and long-term MRI findings and clinical efficacy. White matter has been shown to be more susceptible to sonication than gray matter^23^; therefore, it is possible that FUS thalamotomy with relatively low peak temperature preferentially causes demyelination rather than necrosis, as was observed in our patient. Further postmortem analyses after FUS are important to clarify neuropathological changes and their correlations with the maximal temperature at the target, MRI findings of the lesion, and clinical efficacy.

This is the first histopathologic analysis of human FUS thalamotomy, and we cannot conclude if findings in our patient are expected pathological changes. As the low maximum temperature is considered a risk factor for recurrence of tremors after FUS thalamotomy^24,25^, we speculate that our patient may have had been at increased risk of recurrence of tremors.

Our study has limitations in terms of generalization. First, the lesion observed may be related to suboptimal peak temperature at the target. Additional studies of lesions associated with optimal peak temperature are needed. Second, neurons were relatively preserved in the patient’s FUS lesion, but we did not compare the neuronal density in the ventrolateral thalamus because only the left hemibrain was studied; the right hemibrain was frozen. Third, the patient had stage IV malignant choroidal melanoma with metastases to the liver, which makes interpretation of his disease course challenging. He developed symptoms of neuropathy 2 months after FUS thalamotomy, probably due to immunotherapy. His malignancy also precluded longitudinal assessments of clinical efficacy and MRI characteristics of FUS thalamotomy.

In summary, pathological assessment of a patient with tremor-dominant PD after FUS thalamotomy revealed a 5-mm lesion in the ventral lateral thalamus, which was characterized by demyelination and abundant lipid-laden macrophages and relatively preserved neurons and axons. Further clinicopathological studies on patients treated with FUS are needed to assess the pathological correlates of optimal and suboptimal FUS parameters and their clinical outcomes.

## Methods

### Ethical approval

The brain autopsy on this patient was performed after the consent of the next-of-kin. The brain bank operates under procedures approved by the Mayo Clinic Institutional Review Board. De-identified studies of autopsy samples are considered exempt from human subject research by the Mayo Clinic Institutional Review Board.

### MR-guided FUS thalamotomy

The procedure for performing an MR-guided FUS thalamotomy has been described previously^9,26^. In brief, the patient was prepared with hair clipping and application of a stereotactic head frame under local anesthesia. The patient was positioned supine with a rubber scalp membrane sealed to the ultrasound transducer (InSightec) in a 3 T MRI system. Volumetric MRI was acquired and fused to the patient’s preoperative MRI and CT scans. The left thalamic target was planned 14.0 mm lateral to the midline, 6.8 mm anterior to the posterior commissure, and 2 mm above the commissural plane. Therapeutic sonications were administered to the target with incrementally increasing energy. Clinical monitoring of the patient was obtained after each sonication. Tremor was assessed in the resting and postural states as well as with finger-to-nose and drawing tasks. Potential neurologic adverse effects were monitored with sensory and motor testing, and there were none.

### Neuropathological assessment

The left hemibrain was fixed in formalin and standardized sections were embedded in paraffin. Regions sampled for histopathologic assessment included six regions of the neocortex, two levels of the hippocampus, a basal forebrain section that includes the amygdala, lentiform nucleus and hypothalamus, anterior corpus striatum, thalamus at the level of the subthalamic nucleus, midbrain, pons, medulla, and two sections of the cerebellum, one including the deep nuclei. Paraffin-embedded 5-μm-thick sections mounted on glass slides were stained with H&E and thioflavin S (Sigma-Aldrich, St. Louis, MO). Braak neurofibrillary tangle stage and Thal amyloid phase were assessed with thioflavin S fluorescent microscopy according to published criteria, as previously described^27–30^. Sections of the cortex, hippocampus, and basal forebrain, and brainstem were immunostained with anti-α-synuclein antibody (NACP; rabbit polyclonal; 1:3000; Mayo Clinic Antibody; formic acid pretreatment)^31^ using IHC Autostainer 480S (Thermo Fisher Scientific Inc., Waltham, MA) and DAKO EnVision™ + reagents (Dako, Carpinteria, CA) to confirm a diagnosis of PD^17^. To characterize the lesion related to FUS, we also performed LFB-PAS stains and immunohistochemistry for activated microglia (CD68; 1:1000; mouse monoclonal; DAKO), homeostatic microglia (IBA-1; mouse monoclonal; 1:3000; FUJIFILM Wako Chemicals USA, Corp., Richmond, VA), astrocytes (GFAP; GA-5; mouse monoclonal; 1:5000; BioGenex, Fremont, CA), phosphorylated neurofilaments (SMI-31; mouse monoclonal; 1:20,000; Covance; Berkeley, CA), non-phosphorylated neurofilaments (SMI-32; mouse monoclonal; 1:1000; BioLegend; San Diego, CA), amyloid precursor protein (APP; mouse monoclonal; 1:1000, Millipore Sigma; Burlington, MA), T-cells (CD3; mouse monoclonal; 1:100; Dako), memory T-cells (CD45/leukocyte common antigen; mouse monoclonal; 1:1000, Dako), and B-cells (CD20; mouse monoclonal; 1:1000; Dako) using the section including the thalamus.

### Reporting summary

Further information on research design is available in the Nature Research Reporting Summary linked to this article.

## Supplementary information

Reporting Summary

## Data Availability

Research data are not publicly available since it is protected health information.
