# Supplementary material for: Neuropathology of Parkinson’s disease after focused ultrasound thalamotomy
Source: NPJ Parkinsons Dis. 2022 May 12;8:59. doi: 10.1038/s41531-022-00319-6 (PMC9098516; doi:10.1038/s41531-022-00319-6)
Supplement: Supplementary file 1 — Reporting Summary [file 41531_2022_319_MOESM1_ESM.pdf]

nature portfolio

Corresponding author(s): Dennis W Dickson

Last updated by author(s): Jan 11, 2022

Reporting Summary

Nature Portfolio wishes to improve the reproducibility of the work that we publish. This form provides structure for consistency and transparency in reporting. For further information on Nature Portfolio policies, see our Editorial Policies and the Editorial Policy Checklist.

Statistics

For all statistical analyses, confirm that the following items are present in the figure legend, table legend, main text, or Methods section.

n/a	Confirmed	
☒	☐	The exact sample size (*n*) for each experimental group/condition, given as a discrete number and unit of measurement
☒	☐	A statement on whether measurements were taken from distinct samples or whether the same sample was measured repeatedly
☒	☐	The statistical test(s) used AND whether they are one- or two-sided<br>*Only common tests should be described solely by name; describe more complex techniques in the Methods section.*
☒	☐	A description of all covariates tested
☒	☐	A description of any assumptions or corrections, such as tests of normality and adjustment for multiple comparisons
☒	☐	A full description of the statistical parameters including central tendency (e.g. means) or other basic estimates (e.g. regression coefficient) AND variation (e.g. standard deviation) or associated estimates of uncertainty (e.g. confidence intervals)
☒	☐	For null hypothesis testing, the test statistic (e.g. *F*, *t*, *r*) with confidence intervals, effect sizes, degrees of freedom and *P* value noted<br>*Give P values as exact values whenever suitable.*
☒	☐	For Bayesian analysis, information on the choice of priors and Markov chain Monte Carlo settings
☒	☐	For hierarchical and complex designs, identification of the appropriate level for tests and full reporting of outcomes
☒	☐	Estimates of effect sizes (e.g. Cohen's *d*, Pearson's *r*), indicating how they were calculated

Our web collection on statistics for biologists contains articles on many of the points above.

Software and code

Policy information about availability of computer code

Data collection	No software was used.
Data analysis	No software was used.

For manuscripts utilizing custom algorithms or software that are central to the research but not yet described in published literature, software must be made available to editors and reviewers. We strongly encourage code deposition in a community repository (e.g. GitHub). See the Nature Portfolio guidelines for submitting code & software for further information.

Data

Policy information about availability of data

All manuscripts must include a data availability statement. This statement should provide the following information, where applicable:

- Accession codes, unique identifiers, or web links for publicly available datasets
- A description of any restrictions on data availability
- For clinical datasets or third party data, please ensure that the statement adheres to our policy

Research data are not publicly available since it is protected health information.

Field-specific reporting

Please select the one below that is the best fit for your research. If you are not sure, read the appropriate sections before making your selection.

☒ Life sciences ☐ Behavioural & social sciences ☐ Ecological, evolutionary & environmental sciences

For a reference copy of the document with all sections, see nature.com/documents/nr-reporting-summary-flat.pdf

Life sciences study design

All studies must disclose on these points even when the disclosure is negative.

Sample size	Not applicable because this study is a single case report.
Data exclusions	Not applicable because this study is a single case report.
Replication	Not applicable because this study is a single case report.
Randomization	Not applicable because this study is a single case report.
Blinding	Not applicable because this study is a single case report.

Reporting for specific materials, systems and methods

We require information from authors about some types of materials, experimental systems and methods used in many studies. Here, indicate whether each material, system or method listed is relevant to your study. If you are not sure if a list item applies to your research, read the appropriate section before selecting a response.

Materials & experimental systems

n/a	Involved in the study	
☐	☒	Antibodies
☒	☐	Eukaryotic cell lines
☒	☐	Palaeontology and archaeology
☒	☐	Animals and other organisms
☐	☒	Human research participants
☒	☐	Clinical data
☒	☐	Dual use research of concern

Methods

n/a	Involved in the study	
☒	☐	ChIP-seq
☒	☐	Flow cytometry
☒	☐	MRI-based neuroimaging

Antibodies

Antibodies used	NACP; CD68; IBA-1; GFAP; SMI-31; SMI-32; APP; CD3; CD45/LCA; CD20.
Validation	NACP is a non-commercial antibody produced at Mayo Clinic. This antibody was described and validated on literature (PMID: 10514406).<br>CD68, CD3, CD45/LCA, and CD20 were purchased from Dako.<br>IBA-1 was purchased from FUJIFILM Wako Chemicals USA, Corp.<br>GFAP was purchased from BioGenex.<br>SMI-31 and SMI-32 were purchased from BioLegend.<br>APP was purchased from Millipore Sigma.<br>Manufacturer websites state antibody validation and publications.

Human research participants

Policy information about studies involving human research participants

Population characteristics	Brain tissue from the patient was provided by Mayo Clinic brain bank.
Recruitment	The brain autopsy on this patient was performed after the consent of the next-of-kin.
Ethics oversight	The brain bank operates under procedures approved by the Mayo Clinic Institutional Review Board. De-identified studies of autopsy samples are considered exempt from human subject research by the Mayo Clinic Institutional Review Board.

Note that full information on the approval of the study protocol must also be provided in the manuscript.
